# Nonossifying fibroma presenting as an aneurysmal bone cyst: a case report

**DOI:** 10.1186/1752-1947-6-407

**Published:** 2012-11-29

**Authors:** Akio Sakamoto, Takeaki Ishii, Yoshinao Oda, Yukihide Iwamoto

**Affiliations:** 1 Department of Orthopaedic Surgery, National Hospital Organization Kokura Medical Center, Kitakyushu, Fukuoka, Japan; 2 Department of Orthopaedic Surgery, Graduate School of Medical Sciences, Kyushu University, Fukuoka 812-8582, Japan; 3 Department of Anatomic Pathology, Graduate School of Medical Sciences, Kyushu University, Fukuoka 812-8582, Japan

## Abstract

**Introduction:**

Nonossifying fibroma is a common fibrous bone lesion in children that occurs in the metaphysis of the long bones of the lower extremities. The lesion rarely leads to aneurysmal bone cyst, which is characterized as a blood-filled space.

**Case presentation:**

We present the case of a 13-year-old Japanese boy with a complaint of discomfort in the thigh and a small, well-defined, osteolytic lesion with cortical thinning located in the medullary space of the distal diaphysis of the femur. At 10-month follow-up, the size of the lesion had increased. Gadolinium-enhanced magnetic resonance imaging failed to detect any solid area. Curettage and bone graft were performed and confirmed a blood-filled cystic lesion. The pathological diagnosis of the cyst wall was that of nonossifying fibroma, suggesting aneurysmal bone cyst as a secondary change. An aneurysmal bone cyst is rarely found secondary to nonossifying fibroma, and the diaphyseal location is atypical for nonossifying fibroma, both of which made diagnosis challenging.

**Conclusion:**

The current case is a reminder to clinicians that, although rare, nonossifying fibroma can be associated with aneurysmal bone cyst, and both can occur in the diaphysis of long bones.

## Introduction

Nonossifying fibroma (NOF) is a common type of benign fibrous lesion that occurs in the metaphysis of the long bones of the lower extremities [1,2]. On plain radiographs, NOF appears as a small, cortically based osteolytic lesion with a thin sclerotic rim. Histologically, NOF is composed of spindle-shaped fibroblasts, multinucleated giant cells, and foamy histiocytes [2]. NOF is typically asymptomatic and the lesion is found incidentally. NOF is considered a developmental bone defect, not a neoplastic lesion, because it is usually self-limiting [1-3].

In contrast to NOF, aneurysmal bone cyst (ABC) is a locally destructive bone lesion [4]. Primary ABC typically occurs during the first two decades of life, and most frequently in the metaphyses of long bones, and less frequently in the spine or flat bones [4]. On plain radiographs, ABC appears as an eccentric and expansile lytic lesion [4]. ABC is considered a reactive process secondary to various precursor conditions, including benign and malignant bone neoplasms [2,5].

We report a rare case of NOF associated with ABC that occurred at the diaphysis of the femur.

## Case presentation

**Figure 1:**
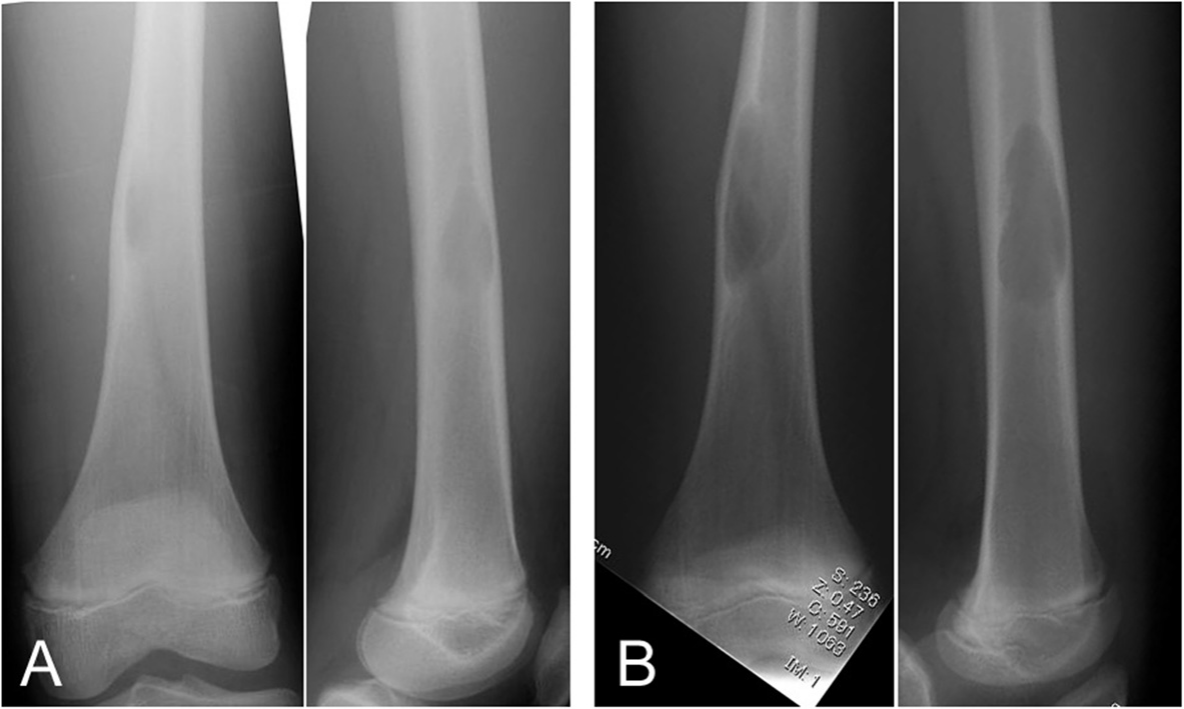
**Antero-posterior (left) and lateral (right) plain radiographs show a well-defined osteolytic lesion in the medullary space with cortical thinning (A).** The size of the lesion increased over 10 months after the initiation assessment on antero-posterior (left) and lateral (right) views (**B**).

**Figure 2:**
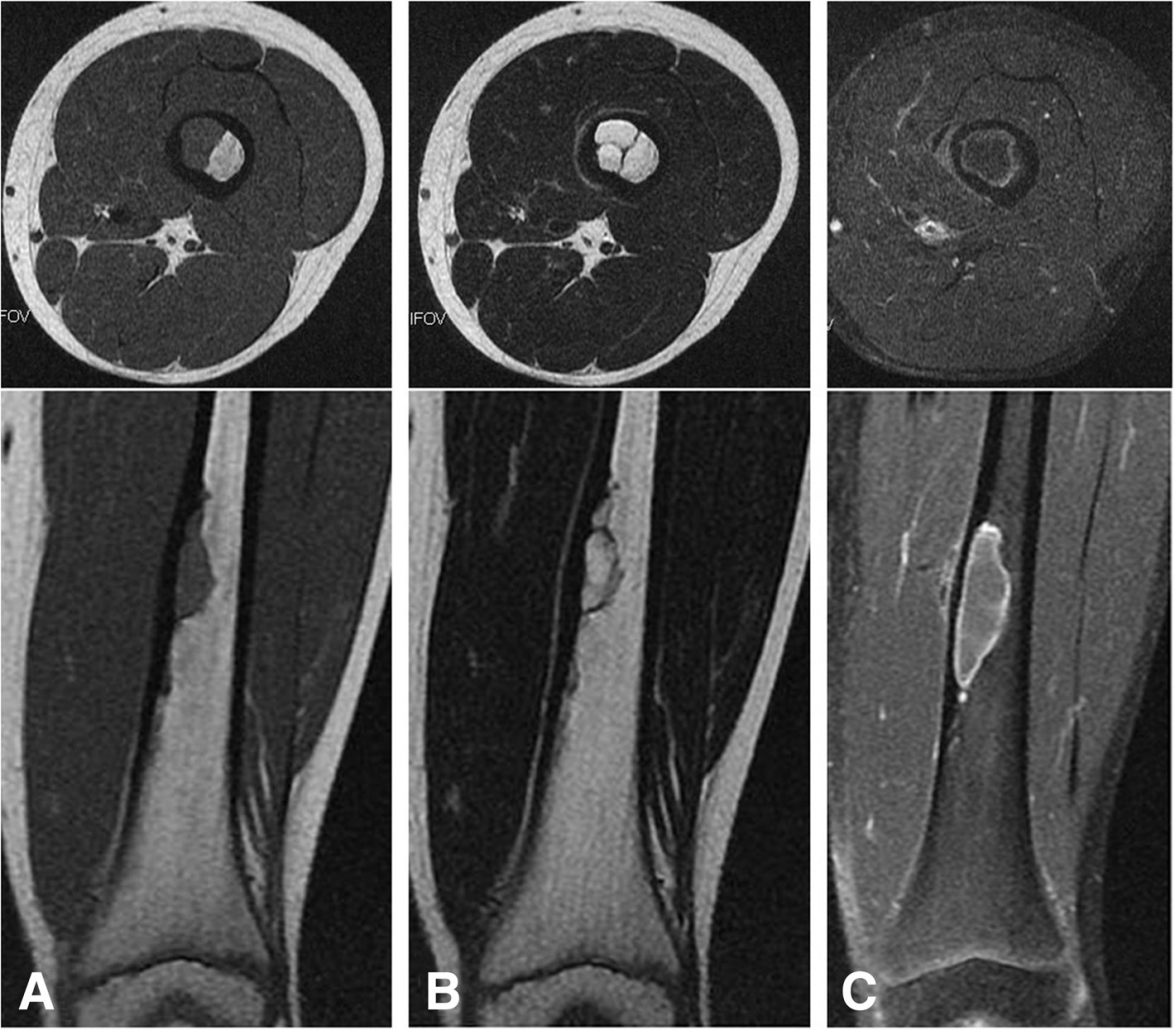
**Magnetic resonance imaging shows a medullary lesion with low signal intensity on (A) T1- and (B) T2-weighted images.** The size of the lesion increased over 10 months (**C**). Enhancement by gadolinium is seen on the periphery of the lesion on the T1-weighted image (**C**).

A 13-year-old Japanese boy with discomfort in his right thigh was evaluated at an orthopedic hospital where an abnormality in the distal diaphysis of the femur was noticed on plain radiographs, and the patient was referred to our institute. The plain radiographs showed a well-defined osteolytic lesion with two-cm longitudinal diameter. The adjacent cortex was slightly expanded and thinned (Figure 1A). Magnetic resonance imaging (MRI) demonstrated the intramedullary lesion with low signal intensity on T1-weighted images and high signal intensity on T2-weighted images. Internal fibrous septa were seen as low signal intensity on both T1- and T2-weighted images (Figure 2A and 2B). There were no signal abnormalities in the intramedullary bone surrounding the lesion, and no extraosseous extension or adjacent soft-tissue abnormality was recognized. Because the features of MRI ruled out aggressive neoplasm or infection, the diagnosis was a benign lesion, and follow-up was recommended.

**Figure 3:**
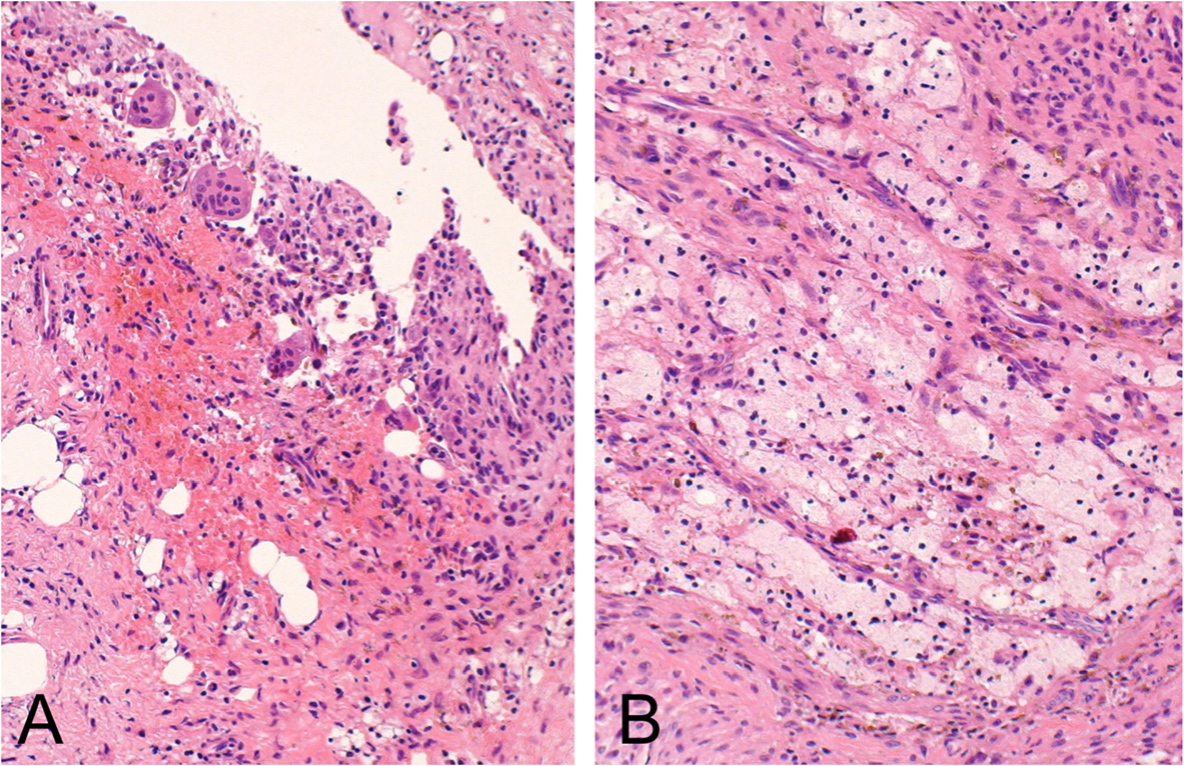
**Nonossifying fibroma.** Histology shows spindle cells arranged in vague fascicles (**A** and **B**) accompanied by hemorrhage and multinucleated giant cells, along with foamy histiocytes.

On follow-up 10 months later, the size of the lesion on plain radiograph had increased to 5cm on longitudinal diameter and MRI confirmed the enlargement (Figures 1B and 2C). MRI with gadolinium contrast enhanced only the periphery of the lesion, a result that suggested the lesion was cystic (Figure 2C). Curettage and artificial bone graft were performed. The gross operative finding was of a cystic lesion containing blood and no solid material. The histology of the curetted tissue from the cyst wall was composed of fibrohistiocytic spindle cells arranged in vague fascicles, accompanied by occasional multinucleated giant cells and foamy histiocytes. The histological diagnosis was that of NOF (Figures 3A and 3B). The final diagnosis based upon the histology, image results, and clinical appearance was ABC change secondary to NOF.

## Discussion

The pathogenesis of ABC is presumably a local circulatory abnormality that causes increased venous pressure and results in dilation of the vascular network [2,5]. Primary ABC has a different genetic pathogenesis from secondary ABC, in spite of their morphological similarities, because primary ABC has been reported to have rearrangements of *CDH11* or *USP6* that are not seen in secondary ABC [6].

Typically, the diagnosis of NOF can be easily made based on images and clinical findings [3,7]. The natural course of NOF is self-limiting involution and, thus, NOF has come to be known as a ‘don’t touch’ lesion because more aggressive diagnostics or treatment are unnecessary [1]. It has been reported that NOF can be found in approximately 30% of young individuals within the first and second decade of life [2]. ABC change secondary to NOF appears to be extremely rare, although the frequency is difficult to assess. Of interest, this case of NOF was located at the femoral diaphysis, but the typical location of NOF is the metaphyseal region. According to Brenner and colleagues [8], NOF can be divided into three phases on the basis of the intensity of uptake on bone scan: active, healing, and inactive. The low uptake, combined with the diaphyseal location, may suggest the current case was a long standing lesion in the inactive phase. The diaphyseal location made it difficult to arrive at the correct diagnosis of NOF based on the imaging results.

## Conclusion

In conclusion, this report presents a rare case of NOF associated with ABC. In addition, the diaphyseal location was atypical for NOF. We present this case report as a reminder to clinicians to remain aware that, although rare, NOF can be associated with ABC, and both can occur in the diaphyses of long bones.

## Abbreviations

ABC: Aneurysmal bone cyst; NOF: Nonossifying fibroma.

## Competing interests

The authors declare that they have no competing interests.

## Authors’ contributions

AS drafted the manuscript. AS and TI treated the patient. YO and YI participated in the design of the study. All authors read and approved the final manuscript.

## Consent

Written informed consent was obtained from the patient’s legal guardian for publication of this manuscript and accompanying images. A copy of the written consent is available for review by the Editor-in-Chief of this journal.
